# Predicting criminality from child maltreatment typologies and posttraumatic stress symptoms

**DOI:** 10.3402/ejpt.v4i0.19825

**Published:** 2013-04-24

**Authors:** Ask Elklit, Karen-Inge Karstoft, Cherie Armour, Dagmar Feddern, Mogens Christoffersen

**Affiliations:** 1 National Centre for Psychotraumatology, University of Southern Denmark, Denmark; 2 Danish Center for Research in Alcohol and Drug Abuse Studies, Aarhus University, Denmark; 3 The National Research Centre for Welfare, Copenhagen, Denmark

**Keywords:** Childhood maltreatment, sexual abuse, emotional abuse, posttraumatic stress disorder, latent classes, criminal behavior, national representative study

## Abstract

**Background:**

The associations between childhood abuse and subsequent criminality and posttraumatic stress disorder (PTSD) are well known. However, a major limitation of research related to childhood abuse and its effects is the focus on one particular type of abuse at the expense of others. Recent work has established that childhood abuse rarely occurs as a unidimensional phenomenon. Therefore, a number of studies have investigated the existence of abuse typologies.

**Methods:**

The study is based on a Danish stratified random probability survey including 2980 interviews of 24-year-old people. The sample was constructed to include an oversampling of child protection cases. Building on a previous latent class analysis of four types of childhood maltreatment, three maltreatment typologies were used in the current analyses. A criminality scale was constructed based on seven types of criminal behavior. PTSD symptoms were assessed by the PC-PTSD Screen.

**Results:**

Significant differences were found between the two genders with males reporting heightened rates of criminality. Furthermore, all three maltreatment typologies were associated with criminal behavior with odds ratios (ORs) from 2.90 to 5.32. Female gender had an OR of 0.53 and possible PTSD an OR of 1.84.

**Conclusion:**

The independent association of participants at risk for PTSD and three types of maltreatment with criminality should be studied to determine if it can be replicated, and considered in social policy and prevention and rehabilitation interventions.

Criminal behavior in adolescence is of great societal concern. Therefore, the identification of risk factors related to criminality has been of great interest. One childhood factor that has been consistently associated with adolescent criminal behavior is childhood abuse (Stewart, Livingston, & Dennison, 2008; Thornberry, Ireland, & Smith, 2001). Notably, a well-established outcome of childhood abuse is psychopathology including posttraumatic stress disorder (PTSD; Claussen & Crittenden, 1991; Deblinger, McLeer, Atkins, Ralphe, & Foa, 1989; Fergusson, Horwood, & Lynskey, 1996). In addition, albeit controversial, studies have reported that psychiatric disorders in general are associated with criminal convictions (Hodgins, Mednick, Brennan, Schulsinger, & Engberg, 1996).

The extant literature has consistently shown significant associations between childhood maltreatment and different forms of criminal and adversive behavior in adolescence. For example, overall delinquency (Heck & Walsh, 2000), violent delinquency (Smith & Thornberry, 1995), juvenile offending (Stewart et al. 2008; Thornberry et al. 2001), and antisocial, aggressive, and violent criminal behaviors (Haapasalo & Pokela, 1999) have all been associated with childhood maltreatment. Some researchers have reported that specific maltreatment types are related to subsequent antisocial development. For example, physical abuse has been found to be independently predictive of violent behavior (Herrenkohl, Huang, Tajima, & Whitney, 2003; Maas, Herrenkohl, & Sousa, 2008; Widom, 1989) and more specifically violent sexual offending (Widom & Ames, 1994). Swantson et al. (2003) reported that children who had experienced sexual abuse were over twice as likely (odds ratio [OR]=2.29) to report involvement in criminal activity compared to non-abused controls. Likewise, Siegel and Williams (2003) reported that children who had experienced sexual abuse were over twice as likely to be arrested as juveniles (OR=2.4) and adults (OR=2.0) compared to non-abused controls.

The experience of childhood abuse has also long been associated with psychopathology. In particular, it is estimated that between 25 and 65% of victims of childhood abuse will go on to develop PTSD (Albach & Everaerd, 1993; Chu & Dill, 1990; Kiser et al. 1991; Palmer et al. 1992). To date, research has predominately focused on the experience of sexual abuse and physical abuse in relation to subsequent psychopathology (cf. Brown & Anderson, 1991; Fergusson, Boden, & Horwood, 2008; Jumper, 1995; Kendler et al. 2000).

Albeit controversial, some studies have reported that psychopathology may also predict criminality. Notably, the prevalence of psychopathology within the prison system far exceeds that of the general population (Brinded et al. 2001). Indeed, Singleton et al. (1998) reported that almost 90% of the prison population has a mental health issue. Furthermore, criminal offenders have been found to possess higher rates of traumatic experiences than non-offenders (Goldenson, Geffner, Foster, & Clipson, 2007) and community samples (Abram, Teplin, Charles, Longworth, McClelland, & Dulcan 2004; Neller, Denney, Pietz, & Thomlinson, 2006). And, the development of violent behavior has been argued to be strongly associated with posttraumatic stress (Begic & Jokic-Begic, 2002).

One notable limitation of research concerned with the effect of childhood abuse is the focus on particular types of abuse at the expense of others (Higgins & McCabe, 2001; Pears, Kim, & Fisher, 2008). Indeed, childhood abuse is commonly divided across domains of physical abuse, emotional abuse, sexual abuse, and childhood neglect (Sedlak et al., 2008). While these categories of childhood abuse are generally agreed upon, no agreement is found when it comes to the relative prevalence of the abuse types. Whereas some studies have found childhood neglect to be the most prevalent type of abuse (U.S. Department of Health and Human Services, 2010), other studies find that emotional abuse is the most prevalent (Armour, Elklit, & Christoffersen, in press; Brooker, Cawson, Kelly, & Wattam, 2001). This might reflect disagreement on the definition and operationalization of child maltreatment within the literature; with different abuse types being difficult to separate. However, it may also reflect a high co-occurrence between the different forms of childhood abuse (Kantor & Little, 2003; Stanley & Goddard, 2004), in that researchers ask questions which do not cover the full spectrum of abuse types or in a reporting bias whereby individuals only report experiencing the particular abuse type which they perceive as their worst, or indeed their most recent experience.

More recently, multiple abuse experiences has been regularly reported within the literature (e.g., Armour et al., in press; Finkelhor et al. 2007; Higgins & McCabe, 2001). However, a less studied area is one which investigates abuse typologies. Armour et al. (in press) conducted a latent class analysis study (using the same nationally representative database as used in this study) which uncovered four abuse typologies, based on how respondents answered to 20 individual indicators of abuse across four different abuse domains. The abuse typologies were characterized as a non-abused group, a predominately psychologically maltreated group, a predominately sexually abused group, and a group experiencing multiple abuse experiences. Membership of abuse typologies was predicted by child protection status (i.e., having been previously known to the Danish social services), female gender was predictive of membership in the predominately sexually abused class and child protection status was predictive of membership in all abuse classes compared to the non-abused class.

The Armour et al. (in press) study did not, however, investigate the association between the abuse typologies and criminal behavior. Nor did the Armour et al. (in press) study investigate the relationship between the abuse typologies and PTSD. Therefore, this study aims to build on the Armour et al. (in press) study by investigating the relationship between childhood abuse typologies, gender, PTSD, and criminal behavior in a nationally representative youth sample.

## Methods

A stratified random probability survey was conducted in Denmark by the National Centre for Welfare between 2008 and 2009. Statistics Denmark randomly selected 4,718 participants, aged 24, from the total birth cohort of Denmark in 1984. Structured interviews were conducted by trained interviewers either in the home or via the telephone. Further details pertaining to the procedure are available in Christoffersen, Armour, Lasgaard, Andersen, & Elklit (in press).

A total of 2,980 interviews were successfully conducted, equating to a response rate of 67%. To increase the number of participants who had experienced childhood abuse and neglect, children who had been in child protection were over-sampled by stratifying the number of “child protection cases” versus “non-child protection cases” (1/3:2/3). A child protection case was defined as a case where the council (according to the files of local social workers) had provided support for the child and the family or placement with a foster family due to concerns about the well-being and development of the child. A total of 852 interviews were conducted with individuals who had been previously identified by the Danish authorities as child protection cases.

The most common reason for non-participation was refusal (21%). Other reasons included illness, disability, and being un-contactable. The sample consisted of 1,579 males and 1,401 females. The majority of the sample either owned or rented their own private accommodation (93.7%) and almost half were married or cohabiting (46.0%). All demographics were analyzed employing a weight variable to account for the oversampling of child protection cases so that findings are representative of the total Danish population of young people aged 24 years. Child protection status (weighted) was given as 6.3% of the total sample.

## Measures

The interview administered a series of questions pertaining to several psychological and physical domains in addition to querying about several demographics. Respondents answered several specific questions across four domains of childhood maltreatment: physical abuse, emotional abuse, neglect, and sexual abuse. For an elaborated description of the measures used to assess child maltreatment, see Christoffersen et al. (in press). As previously noted, the abuse typologies were derived by Armour et al. (in press) who implemented the statistical technique of latent class analysis. Four abuse typologies were uncovered from 20 separate indicators of abuse spanning four abuse domains: the predominately psychologically maltreated group (8.8%), the predominately sexually abused group (2.0%), the overall abuse group (physical abuse+neglect+emotional abuse; 2.1%), and the non-abused group (87.1%).

Criminal behavior was assessed by seven items questioning whether an individual had ever shoplifted, stole a bicycle, stole a car, committed burglary, vandalism, or violence, or been convicted of a crime (Christoffersen, 1996; Kyvsgaard, 1992). Respondents answered “yes” or “no” to these seven items (Table 1). A scale was computed with these seven items (Cronbach's alpha=0.75). Criminality was endorsed if scores were ≥3, which was the case for 10.9% of the sample. Four items (stealing a car, burglary, violence, and conviction) were endorsed by 69–96% of the ≥3 criminality group; the three other items were endorsed by 38–45% of the same group. This indicates that the cut-off presents well as a criterion for more serious crime.

**Table 1:** Prevalence of the criminal behavior by gender

	Total	Male (%)	Female	% high criminality endorsement (≥3)
Shoplifting	619	378 (61)	241	38
Theft of a bike	739	585 (79)	154	40
Theft of a car	68	63 (91)	5	96
Burglary	108	101 (95)	7	89
Vandalism	663	565 (85)	98	45
Violent assault	265	224 (85)	41	69
Convicted	159	147 (92)	12	86
Total	2,621	2,083 (79)	558	
Number≥3	326	291 (89)	35	

Screening for possible PTSD was assessed using the Primary Care PTSD Screen (PC-PTSD; Prins et al., 2003) with four dichotomous items representing the main symptom groups, including intrusive memories, avoidance, increased arousal, and emotional numbing. A score ≥3 was determined to be the most efficient cut-off in U.S. adult primary care samples and is used to identify participants with likely PTSD (Prins et al., 2003).

### Data analysis

The relationship between childhood abuse and criminality was tested by a logistic regression model using criminality as the dependent, categorical variable and exploring the predictive ability of the childhood abuse typologies, gender, and PTSD. The forced entry method was chosen as all predictor variables are tested in one block to assess their predictive ability, while controlling for the effects of other predictors in the model.

## Results

Descriptive statistics on the prevalence of criminal behavior by gender can be seen in Table 1. All differences were significant (all *F*s>25; all *p’*s<0.0005) by a one-way ANOVA test. Table 2 shows criminality scores by abuse classes and by gender endorsement. Sexual abuse has an endorsement a little less than the non-abused class. The overall abuse and the emotional abuse classes have comparatively large ORs; the same is the case for sexual abuse but one has to be aware of the very small *n* in this particular class.

**Table 2:** Abuse classes by gender endorsement

Allocation	High criminality Score *N* (%)	Low criminality score *N* (%)	Odds ratios	95% CI
All				
Emotional abuse	71 (22)	192 (7)	4.25	3.04–5.95
Sexual abuse	5 (2)	55 (2)	0.64	0.24–1.71
Overall abuse	22 (7)	42 (2)	5.99	3.44–10.45
Non-abuse	229 (70)	2,366 (89)	0.26	0.19–0.34
Total *n*	327	2,655		
Male				
Emotional abuse	62 (21)	72 (6)	5.76	3.78–8.77
Sexual abuse	1 (0)	1 (0)	5.40	0.31–94.66
Overall abuse	17 (6)	12 (1)	10.22	4.47–23.38
Non-abuse	210 (72)	1,179 (93)	0.15	0.10–0.22
Total *n*	290	1,264		
Female				
Emotional abuse	9 (25)	119 (7)	3.90	1.64–9.27
Sexual abuse	4 (11)	54 (4)	2.67	0.84–8.52
Overall abuse	4 (11)	29 (2)	8.23	2.75–24.67
Non-abuse	19 (53)	1,187 (87)	0.18	0.09–0.36
Total *n*	36	1,389		

A logistic regression was performed for the three abuse classes, gender, PTSD, and their relation to criminal behavior to estimate the predictive value of these variables (see Table 3). The Omnibus Tests of Model Coefficients and the Homer and Lemeshow test both supported the model and indicated a goodness of fit. All variables contributed significantly to the predictive ability. All three abuse classes were significantly associated with criminal behavior with odds ratios (ORs) varying from 2.90 to 5.32, sexual abuse having the lowest, followed by emotional abuse and overall abuse having the highest ORs. Gender (being female) was a predictive factor with an OR of 0.53. Controlling for all other factors, PTSD added independently to the prediction of criminal behavior with an OR of 1.84. The regression model accounted for between 12% (Cox & Snell) and 24% (Nagelkerke) of the variation in criminal behavior.

**Table 3:** Logistic regression analysis predicting criminal behavior from three classes of childhood maltreatment, gender, and PTSD

							95% CI	
							
Predictor	B	SE	Wald	*df*	Sig.	OR	Lower	Upper
Constant	−1.25	0.10	149.8	1	0.001	0.28		
Emotional abused	1.35	0.18	57.6	1	0.001	3.85	2.72	5.46
Sexual abused	1.06	0.54	3.9	1	0.05	2.90	1.01	8.31
Overall abused	1.67	0.33	25.8	1	0.001	5.32	1.80	10.13
Gender	−0.65	0.05	161.5	1	0.001	0.53	0.48	0.58
PTSD	0.61	0.10	34.1	1	0.001	1.84	1.50	2.25

Final model, *X*^2^ (5)=372.5; *p*=0.001. *R*^2^=0.12 (Cox & Snell), 0.24 (Nagelkerke).

## Discussion

A significant positive relationship between child abuse and criminal behavior has been found rather consistently in the literature. In this study of a large, representative Danish youth sample, we found several significant associations between all three classes of childhood maltreatment and criminal behavior. Furthermore, we found substantial independent effects of both gender and PTSD symptomatology on criminal behavior, controlling for the effects of abuse classes.

The relationships observed in this study were quite strong. The study extends the extant research by using empirical based abuse classes and not isolated, constructed abused types. In line with the extant research in the field (Siegel & Williams, 2003; Stewart et al. 2008; Swantson et al. 2003; cf. Thornberry et al. 2001), we found that all abuse typologies were associated with criminal behavior to various degrees. Indeed, the associations between classes and criminal behavior appear to be theoretically meaningful. The overall abused class, who strongly endorsed experiences of physical abuse, emotional abuse, and physical neglect, had the strongest risk for criminal behavior later in life. This is supported by literature related to the cumulative effects of trauma. Indeed, studies have shown that cumulative trauma can exacerbate psychopathology (Shevlin, Houston, Dorahy, & Adamson, 2008); thus, we can speculate that cumulative trauma in the form of multiple types of childhood abuse experiences also has the ability to exacerbate alternative negative outcomes such as criminality. Those who were predominately emotionally maltreated in childhood also had a high risk of later criminal behavior and the same was the case, although to a lesser degree for the group who were sexually abused.

Independent of abuse class, female gender appeared to be a protective factor, and this was expected based on the abundance of research which indicates that males are more likely to participate in criminal acts or display criminal behaviors compared to their female counterparts (Steffensmeier & Allan, 1996). Thus, it stands to reason that being female will decrease the likelihood with which an individual is criminal. Criminogenic theories suggest that females are more likely *not* to partake in criminal activity because gender norms, social control, lack of physical strength, and moral and relational concerns restrict female access to criminal *opportunity*. The same factors also limit female willingness to participate in crime at the *motivational* level. In addition, at a *contextual* level there are typical gender differences, females often being involved in simple forms of delinquency, and are unlikely to use weapons or intend serious injury to their victims (Steffensmeier & Allan, 1996). The interesting aspect of the current study is that we have shown that this association holds even when controlling for the experience of abuse and PTSD.

Those who suffered from PTSD due to childhood abuse or due to other traumas later in life had almost a double risk of criminality. This is supported by Loeber, Farrington, Stouthamer-Loeber, Moffitt, and Caspi (1998) who laid the ground for a developmental criminology that suggests that offending has both long-term and immediate antecedents. They view serious offending as caused by the accumulation of deviance processes from childhood through to adulthood. The basic premise of the theory is that offending results from forces within the individual (impulsivity, lack of guilt) and forces in the social environment (parental rearing practices, rejection by peers) in different contexts (work place, home, neighborhood). All three abuse classes are examples fitting well to this theory as forces in the social environment and the chronic PTSD state is an example of forces from within the individual, which represents a vulnerability that is often turned inwards (particularly in females) but can also be turned outwards in acting-out behavior (especially in males; Miller, Kaloupek, Dillon, & Keane, 2004).

This study had several limitations. First, the study was cross-sectional and relied on self-report. Given that the abuse events have occurred many years earlier, this might cause a certain bias, potentially leading to over- or under-reporting. Also, current psychosocial functioning could bias the recall of prior abuse. The PC-PTSD Screen is proxy for PTSD and does not cover all symptoms of the diagnosis. Likewise, the inclusion of specific abuse events before the latent class analysis was performed does not rely on an established consensus. The same can be said of the constructing of the criminality measure and the cut-off points chosen. The small *N* for sexual abuse in the multivariate regression analysis also demands caution against concluding that sexual abuse exposure is associated with criminal behavior. The found association should be tested in other studies with larger samples.

The findings on the long-term effects of the three abuse classes and PTSD deserve further investigation. The relationship between childhood abuse and criminal behavior should be further investigated with other measures to find more mediating or moderating current variables like alcohol and drug abuse, cognitive mechanisms, peer and parent relationships, attachment style, personal resources, and others. In addition, longitudinal studies could provide important historical information about critical time periods, parental functioning, revictimization, etc.

In conclusion, this study demonstrates the independent association of possible PTSD with self-reported criminal behavior, controlling for the effects of childhood abuse and gender based on a large representative national sample. Combining childhood abuse assessment with screening for PTSD strengthens the possibilities for an informed early intervention which could aid in the prevention of young people entering a life of criminality.
